# Dental Anxiety and Oral-Health-Related Quality of Life among Rural Community-Dwelling Older Adults

**DOI:** 10.3390/ijerph19137643

**Published:** 2022-06-22

**Authors:** Bothaina Hussein Hassan, Maha Mohammed Abd El Moniem, Shaimaa Samir Dawood, Abdulrahman Abdulhadi Alsultan, Amal Ismael Abdelhafez, Nancy Mahmoud Elsakhy

**Affiliations:** 1 Department of Nursing, College of Applied Medical Sciences, King Faisal University, Al-Ahsa 31982, Saudi Arabia; aabdelhameed@kfu.edu.sa; 2 Department of Gerontological Nursing, Faculty of Nursing, Alexandria University, Alexandria 21527, Egypt; maha.mohammed@alexu.edu.eg (M.M.A.E.M.); shaimaa.dawoud@alexu.edu.eg (S.S.D.); 3 College of Medicine, King Faisal University, Al-Ahsa 31982, Saudi Arabia; aalsultan@kfu.edu.sa; 4 Department of Critical Care & Emergency Nursing, Faculty of Nursing, Assiut University, Asyut 71717, Egypt; 5 Department of Gerontological Nursing, Faculty of Nursing, Matrouh University, Marsa Matrouh 51511, Egypt; nancy_elsakhy@mau.edu.eg

**Keywords:** dental anxiety, dental care, oral health, quality of life, older adults, rural, community-dwelling

## Abstract

**Aim:** This study aimed to investigate the association between dental anxiety (DA) and oral-health-related quality of life (OHRQoL) among rural community-dwelling older adults. **Methods:** A cross-sectional descriptive study was conducted among 390 rural community-dwelling older adults attending outpatient clinics of the Damanhur National Medical Institute during the year 2021. Three instruments were used: a demographic and clinical data structured interview schedule, Modified Dental Anxiety Scale (MDAS), and the Oral Health Impact Profile (OHIP-5) questionnaire. All statistical analyses were considered significant at a *p*-value of ≤0.001. **Results**: The main results showed that the prevalence of DA among participants was 90.5%, and 66.9% of the studied elderly population were either extremely (phobic) or very anxious. Moreover, a significant association was found between older adults’ DA and their OHRQoL (*p* ˂ 0.001). **Conclusions:** It was concluded that DA represents a common problem among rural community-dwelling older adults and is a predictor for poor OHRQoL. Raising community awareness about the importance of oral health and implementing measures to avoid DA through specialized community campaigns is recommended, particularly in rural areas.

## 1. Introduction

Oral health care distribution, accessibility, utilization, and outcomes varied significantly between rural and urban locations in both developed and developing countries, including Egypt. The impoverished and disadvantaged inhabitants of rural regions bear the brunt of oral illness worldwide [1,2,3,4]. People in rural locations are more likely to be poorer, are less health-literate, have more caries, and have a more limited budget for dental treatment than people in metropolitan areas [4]. Health insurance in Egypt is frequently linked to employment; the majority of older people in rural areas lack insurance and cannot afford dental procedures [4].

According to the World Oral Health Report 2003, oral diseases are age-related, with most oral diseases sharing risk factors with chronic diseases, and oral health is an important component of overall health and quality of life (QOL) [5]. However, when discussing health issues among the elderly, oral health is frequently overlooked [6,7,8].

A high amount of tooth loss, dental caries, periodontal diseases, dry mouth, and the chance of developing oral precancer/cancer were the most evident signs of poor oral health in the elderly [6,8,9]. The link between oral health and overall health is especially pronounced in the elderly. Poor oral health can worsen general health risks and reduce nutritional intake due to impaired chewing and eating abilities. Similarly, the high prevalence of multi-medication therapies in this age group may complicate the impact on oral health even further. Pain, dental abscess endurance and eating and chewing problems, as well as embarrassment about the shape of teeth or missing, discoloured, or damaged teeth, can all have a negative impact on people’s daily lives and well-being [5,10]. As a result, this may have a detrimental influence on older individuals’ oral-health-related quality of life (OHRQoL), since it makes fundamental daily requirements, such as chewing food and communicating more difficult. This leads to physical and psychosocial health issues such as poor nutrition, low self-esteem and social inadequacy [10]. Given the deleterious impact of poor oral health on health and OHRQoL, addressing oral health care for older persons is a critical public health issue that policymakers must consider [11]. Anxiety over dental services and treatment, on the other hand, can be a significant impediment to achieving good oral health [12].

Dental anxiety (DA) is a negative psychological reaction to stress that is specific to dental situations [12,13]. It is a global public health concern that affects people of all ages and from all geographical locations, and has an impact on people’s oral health and quality of life [14]. In the literature, the relationship between age and level of DA is still unclear, and researchers have suggested contradictory results [15]. In this context, the current study further investigates dental anxiety in rural community-dwelling older adults and its association with OHRQoL. As a result, we can add to the body of knowledge about DA among older adults.

Variety of factors can contribute to dental anxiety [16]. Patient factors include unfavorable or traumatic events in the past, pain, family or peer pressure, and personality qualities, whereas health professional factors include a communication gap and/or unprofessional behavior. An individual’s reaction to seeing needles or blood, the sound of drilling or the presence of other anxious patients, an unpleasant odor/clinic area, the vulnerable position of resting in a dental chair, and the injection of local anesthesia are all examples of environmental factors [12,17].

Dental anxiety causes a vicious cycle of dental avoidance, in which dentally anxious individuals have fewer dental visits, more appointment withdrawals, and a greater proclivity to self-medicate [12,18,19], resulting in a disruption in eating and daily living habits due to pain and dysfunction. They engage in obvious social avoidance behaviors as a result of their dissatisfaction with the appearance of their teeth [20,21]. Otherwise, dentally anxious people can generate significant stress for health care providers due to challenging management and time-wasting [12].

Previous research had discovered a link between DA and OHRQoL [20,22,23,24], but this research has been focused on younger people. Furthermore, studies of elderly people in both institutional and noninstitutional settings have discovered that socioeconomic status, dental disease, regular dental visits, treatment-seeking behaviour, impairment of normal daily activities, degree of systemic disease and self-perceived oral health are among the main factors affecting their OHRQOL [25,26]. In addition, in medically compromised older people, factors like missing teeth, dry mouth and chewing difficulties have been linked to lower quality of life scores [27]. However, little research has been done on the relationship between DA and OHRQoL in older adults [28]. Unfortunately, there are few published data on DA among the Egyptian rural older population, despite the fact that this unique population has significant dental health disparities and limited access to dental care providers, all of which would affect their OHRQoL [2,28]. As a result, this research was designed to investigate the association between dental anxiety and oral-health-related quality of life among rural community-dwelling older adults.

## 2. Materials and Methods

### 2.1. Ethical Consideration

The Ethical Review Board of the College of Applied Medical Sciences, King Faisal University, Saudi Arabia, gave their approval for the study to be conducted (January 2021). Written informed consent was obtained from each participant. The consent form contained the following information: research title, authors contact information’, purpose of the study, subject participation, potential risks and discomforts, potential benefits, compensation, and voluntary participation. Participants’ anonymity was respected, and the confidentiality of the obtained data was assured.

### 2.2. Research Design and Participants

A cross-sectional descriptive research design was used in this study to investigate the association between dental anxiety and oral-health-related quality of life among rural community-dwelling older adults. Three hundred and ninety participants were conveniently recruited from 19 outpatient clinics affiliated with the Damanhur National Medical Institute, which serve the population residing Damanhur city and its associated villages in El-Beheira Governorate. To be eligible, study participants had to reside in one of the rural areas of Damanhur center, be aged at least 60 years old (retirement age), able to communicate, and willing to participate in the study.

***Sample size:*** The study sample size was calculated by the sample size calculator website using the following parameters: population size: 38,000, population proportion: 50%, confidence level: 95%, margin of error: 5%, and minimal sample size: 381. This number was rounded up to the next round number (390).

### 2.3. Measured Outcomes

Initially, the researchers created an Older Adults’ Socio–Demographic and Clinical Data Structured Interview Schedule based on a review of the relevant literature. This was composed of three parts: Part 1: socio-demographic data such as age, sex, marital status, educational level, and monthly income; Part 2: Clinical data, e.g., medical diagnosis oral health problems, and smoking history; Part 3: Oral health practices, such as oral hygiene, pattern of visits to the dentist, and perception of oral health value.

In addition to the first tool, the authors used two standardized instruments, the Modified Dental Anxiety Scale (MDAS) and the Oral Health Impact Profile-5 (OHIP-5) questionnaire. The MDAS was created in 1995 [29] as an enhancement of Corah’s Dental Anxiety Scale (CDAS) [30]. The MDAS involves five questions that assess DA levels through an assessment of the person’s subjective reaction to various dental situations. For each question, there are five responses on a Likert scale from 1 to 5, where 1 represents “not anxious” and 5 means “extremely anxious”. The answers to all five questions are added together to determine the patient’s level of dental anxiety, yielding a total score of from 5 to 25: 0–5 (not anxious), 6–10 (low anxiety), 11–14 (moderate anxiety), 15–18 (high anxiety), and 19–25 (extreme anxiety/phobic) [31]. The MDAS has been translated into several languages [32], and has a high level of cross-cultural validity and reliability [33]. The researchers used the Arabic version of the scale to conduct this study [34], and it was found to be reliable and valid in previous studies [35,36].

The ultra-short version of the Oral Health Impact Profile-5 (OHIP-5) questionnaire [37] was used to assess OHRQoL, which consists of 5 key questions. The English version of the tool was translated into Arabic language and its back-translation was verified by a panel of gerontological nursing experts. For every OHIP-5 question, the participants were asked how often they had encountered the problem in the previous month. The responses were graded on a scale from 1 to 5, where 1 represents “never” and 5 represents “very often.” The OHIP-5 summary score ranged from 5 to 25, with higher numbers indicating poorer OHRQoL.

***Reliability Measures:*** Cronbach’s alpha coefficient was utilized to assess the reliability of tools II (Modified Dental Anxiety Scale (MDAS)) and III (Oral Health Impact Profile (OHIP-5)) questionnaire. Tool II reliability was 0.902, while tool III reliability was 0.847.

### 2.4. Procedures and Data Collection

Prior to the actual study, a pilot study was carried out on 20 older adults who were then excluded from the study sample. The researcher introduced herself to the eligible participants during their waiting time at the outpatient clinics’ waiting area while sitting comfortably. Once older adults expressed their voluntary willingness to participate in the study after a detailed explanation of the study aim and objectives, the participants proceeded to anonymously complete the structured interview with the researcher. The research data were collected by a single trained researcher, who verified the data reported by the patients regarding the presence of oral health problems. The data were collected between the end of June and the middle of December 2021.

### 2.5. Statistical Analysis

The IBM SPSS software program version 20.0 was utilized to analyze the data that were fed into the computer (IBM Corporation, Armonk, New York, NY, USA). Numbers and percentages were used to describe qualitative data. The range (minimum and maximum), mean and standard deviation were used to describe quantitative data. The F-test (ANOVA) was used to compare two or more groups of normally distributed quantitative variables. Moreover, Pearson’s correlation coefficient was utilized to assess the direction and strength of the relationship among older adults’ OHRQoL and MDAS. At a *p*-value ≤ 0.001, all statistical analyses were considered significant. The OHIP-5 total score was categorized as either “poor” or “good” based on the median of 17. Those with scores below 17 were considered “good”, while those with scores equal to or higher than 17 were considered “poor”. Binary logistic regression was performed, and sex was included as a confounder in the model. Odds ratios (ORs) with 95% CIs were also obtained.

## 3. Results

The current study included 390 older adults with a mean age of 1.65 ± 2.4 years. A total of 60.5% were male, 43.6% attained higher education, and 60.3% were married. Regarding dental care expenditure coverage, only 10.3% of the studied participants had health insurance coverage. Hypertension was at the top of the list (63.1%) of common chronic health problems reported by the studied subjects, followed by diabetes mellites and respiratory diseases, at 29.0% and 13.8%, respectively. Tooth loss was reported by 73.3% of the studied older adults, while dental plaque was reported by 10.5% (Table 1).

Table 2 shows that 44.4% of the studied older adults did not clean their teeth. Nearly two thirds (62.3%) of the studied subjects visited the dentist only in emergency situations. Some (12.3%) of the subjects were dissatisfied with their previous dental visits. Long waiting times (47.9%), inconvenient appointments (31.3%), and lack of respect for the elder’s dignity (27.1%) were the most reported reasons for dissatisfaction among older adults. Regarding the perception of oral health value, more than three quarters (77.9%) of the studied older adults perceived their oral health as being a part of their general health. In addition, 66.9% of the studied subjects perceived that they have a moderate oral health condition. The most common reasons for dental anxiety/fear of dental visits among the studied elders, after exclusion of those who had no dental fear (9.4%), were fear of infection, fear of bleeding, embarrassment over tooth shape, and pain felt during dental procedures (76.2%, 68.5%, 61.5%, and 60.0%, respectively).

The results of this study demonstrate that the most affected areas of OHRQoL were complains of less flavor in food (36.7% reported this problem often), and difficulty completing usual jobs because of problems with mouth, teeth, dentures, or jaws, as reported by 36.2%. Moreover, the overall mean score of the OHIP-5 was 15.6 ± 4.6 (Table 3).

Table 4 reveals that 39.5% of the studied elderly population were extremely anxious (phobic), 27.4% very anxious, and 13.6% slightly anxious, while only 9.5% of them were not anxious.

**Figure 1:**
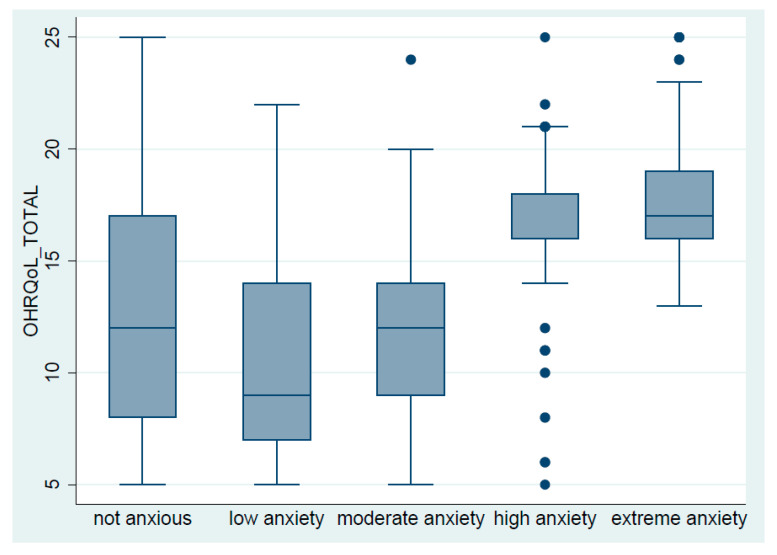
Oral-health-related quality of life by dental anxiety sub-groups.

Table 5 shows a statistically significant difference in OHIP-5 mean scores according to the MDAS score (*p* ≤ 0.001). Moreover, a post hoc analysis of DA subgroups demonstrated that not anxious versus highly and extremely anxious subgroups are statistically different in terms of their overall OHRQoL. Similarly, the low anxious subgroup versus highly and extremely anxious were statistically significant different (Figure 1).

Table 6 revealed a moderate positive correlation between the total MDAS score and total OHIP-5 scale score (*p* ˂ 0.001).

Table 7 shows that those with a low level of DA were 3.75 times more likely to have good OHRQoL compared to those who were not anxious when controlling for the effect of sex. Similarly, those with a moderate level of DA were 3.03 times more likely to have good OHRQoL compared to those who were not anxious when controlling for the effect of sex. Ironically, those with high and extremely high levels of DA were 86% and 83% less likely to have good OHRQoL compared to those who were not anxious when controlling for the effect of sex. Females are two times more likely to have high OHRQoL compared to males while controlling the effect of dental anxiety and age. Those who belonged to middle age category were 38% less likely to have good OHRQoL compared to younger old while controlling for dental anxiety and sex.

## 4. Discussion

In recent decades, the psychological impact of dental disorders on daily life has received more attention, with subjective oral health indices being used to analyze the impact of dental and oral conditions on various populations [38]. To our knowledge, this is the first study in Egypt to assess oral-health-related quality of life and dental anxiety in older people living in rural communities.

The current research found a strong association between dental anxiety and oral-health-related quality of life, with a greater DA score being associated with a lower OHRQoL. Furthermore, our study findings proved that high and extremely levels of anxiety were predictors of poor ORHQoL using the binary logistic regression analysis. In a similar vein, increased DA has globally been linked to poor OHRQoL for patients in Canada [39], Netherlands [24], Hong Kong [40], India [23], Switzerland [22], and Sweden [20]. Another study published in 2018 [41] found that 170 people with severe DA had a low OHRQoL, and this was linked to the prevalence of dental pain. Furthermore, the findings of an epidemiological cross-sectional study [42] among middle-aged women in 2012 revealed that high DA and a lack of sense coherence predicted poor oral-health-related quality of life. Our findings agreed with a study [43] conducted in 2021 to investigate the relationship between DA and OHROL among 118 Saudi elderly individuals, which found a strong link between dental anxiety and poor oral-health-related quality of life. In contrast, a study performed in India in 2019 found no statistically significant relationship between OHRQoL and dental anxiety or oral health status [44].

Furthermore, the current study clarified that the overall OHRQoL significantly changed according to the level of dental anxiety. To deeply investigate the association between DA and OHRQoL, the binary logistic regression analysis was conducted while controlling for the effect of sex and age. This revealed that low and moderate levels of DA had a protective positive impact on OHRQoL, whereas high and extremely high levels had a negative impact. This is considered an effect measure modification, and this new finding tries to challenge the previous studies and requires further investigation. Our study findings were in line with a study [21] that associated DA with OHRQoL (*p* < 0.01) while controlling for socio-demographic factors and oral health status factors, and found that those with high levels of dental anxiety (DA ≥ 15) were approximately two times as likely to be among those experiencing the poorest OHRQoL (below the population median OHRQoL-UK (*W*)^©^ score) in Britain (*p* < 0.001; OR = 1.93; 95% CI 1.41, 2.65). Another study [20] that assessed the relationship between DA and OHRQoL compared the high- and low-DA groups and found that the prevalence of low OHRQoL was 7.7 % in the low-DA group and 24.3% in the high DA group. Mehrstedt et al. [45] confirmed that the median OHIP score of patients with phobic DA was much higher than those observed for general population subjects, indicating that the degree of impairment in OHRQoL is related to the extent of dental anxiety.

Dental anxiety remains a significant barrier to dental treatment, as shown in a study [46] conducted among the Norwegian adult population, which found a link between DA and avoiding dental appointments. Declining dental health in older adults can lead to serious general health problems [47]. This can be a source of insecurity, low self-esteem, social isolation, and, consequently, depression and other serious psychosomatic and psychiatric conditions [48]. According to our findings, nearly two-thirds of older adults sought a dentist’s help only if they had an urgent oral problem. This could be due to their low income and the high cost of dental visits (63.1% of the studied participants reported not having enough income, and only 10.3% had health insurance coverage), as well as depending on others to drop them off to the clinic because they are old. Our justifications were in line with a study [49] that investigated older people’s experiences with oral care and discovered that they considered dental visits to be too expensive and of lesser importance.

Our findings also revealed that 12.3% of the participants were dissatisfied with their previous dentist visits. A similar finding was found by Sgan-Cohen et al. [50] in their 2004 study, which surveyed 162 geriatric patients who had completed a curative course in an oral rehabilitation clinic. Cohen et al. further confirmed that higher levels of satisfaction with the dental team may contribute to higher levels of oral health. He also added that patients who are dissatisfied with their dental care may avoid going to the dentist, putting their health at risk.

According to the literature, DA is thought to start in childhood, peak in early adulthood, and then decline as people get older [51]. However, in our study, more than one half of the elders reported having various levels of dental anxiety, where 39.5% and 27.4% of the participants had extreme (phobic) and very anxious levels of dental anxiety, respectively. In line with the current study, a previous study [28] measured DA among 645 rural older adults who reported high dental anxiety. On the other hand, Mohammed et al. [52] found that severe anxiety (phobia) was uncommon among study participants. The discrepancies between our findings and those of the other studies could be due to the following reasons: painful or traumatic childhood dental experiences and long-term dental anxiety attitudes toward dental services, which make family members or friends afraid. The reasons for dental anxiety/fear of dental visits were analyzed in our study, with the most common reported reasons being fear of infection, fear of bleeding, embarrassment over tooth shape, and pain felt during dental procedures. This result was congruent with another study conducted in Saudi Arabia [53], which identified anesthetic gum injection as the most common source of DA among their participants and elaborated a stronger association between a high dental anxiety score and fear of bleeding of the gums.

Oral health is a reflection of the overall body health. Oral health needs to be approached with caution in older adults, who typically undo physiologic changes in their oral cavity. This is a normal part of the aging process and will affect their OHRQoL [54]. In the current study, the overall mean of OHIP- 5 score was 15.6 ± 4.6, indicating that the participants had poor OHRQoL. This finding may be explained and verified by another finding in our study; approximately three-quarters of the current study’s elderly experienced tooth loss and more than two thirds experience tooth decay, which will definitely decrease the participants’ OHRQoL. This was in line with the study findings [55], which confirmed that missing teeth have a negative impact on oral-health-related quality of life. This was also similar to a study that found that participants who had lost their teeth had showed greater oral impairment and poor oral health quality [56]. Our findings provided an additional insight into how OHRQoL was affected, as it was noted that the most affected areas of OHRQoL were complaints of less flavor in food and difficulty performing usual jobs due to problems with teeth, dentures, mouth, or jaws. Identifying the association between dental anxiety and OHRQoL can provide guidance in many ways: helping policy makers to develop dental care policies [57] and helping in the formulation of anxiety treatment plans that help to improve oral health status, reduce dental anxiety, and enhance OHRQoL [58].

The findings of this study should be interpreted within the context of its limitations. First, because this study’s sample was not a stratified cluster sample, it may represent a mere snapshot of the entire population, limiting its generalizability. Second, we acknowledge that the study depended on self-reported instruments of various oral health outcomes, which could lead to biases in the collected data. Nonetheless, this study had several strengths, including a large sample size from rural community-dwellers, standardized measures of OHRQoL and dental anxiety. Furthermore, the data were collected by a single investigator, ensuring consistency in the questions that were asked to the participants.

In summary, dental anxiety contributes to poor OHRQoL. To facilitate and enhance the intervention process, dental anxiety, as well as subsequent avoidance and neglect behaviors, must be addressed. It is necessary for health care team members, including gerontological nurses, to have a thorough understanding of dental anxiety in order to provide appropriate and effective management. The focus and resources should then be directed toward identifying and managing dentally anxious older people in order to help them maintain better oral health and, thus, a higher quality of life.

## 5. Conclusions and Recommendations

The findings of the present study revealed an extreme level of dental anxiety (dental phobia) among rural community-dwelling older adults. The study further proved a significant association between the older adults’ dental anxiety and their oral-health-related quality of life, and DA was a predictor for poor OHRQoL. Thus, the study recommends an assessment of older adults’ dental anxiety by using valid and reliable tools as an integral part of the periodic dental assessment to ensure early detection and proper management of dental anxiety, and consequently improve their OHRQoL. Future replications of this study are needed using a qualitative research design, which will deepen our understanding of DA among older adults. Further research studies are needed to compare between urban and rural communities in relation to DA and OHRQoL. Community awareness of the importance of oral health and measures to avoid dental anxiety should be raised through specialized community campaigns, especially in rural areas. Finally, when developing a management plan to reduce DA in older adults, management should be tailored to each elder and should be based on the level of anxiety, patient intellect, age, gender, educational level, elders’ perception of oral health, and clinical situation.

## Figures and Tables

**Table 1:** Older adults’ sociodemographic and clinical data.

Sociodemographic and Clinical Data	No. (390)	%
**Sex**		
Male	236	60.5
Female	154	39.5
**Age**		
60-	339	86.9
70-	44	11.3
80+	7	1.8
Mean± SD	65.1 ± 4.2 years	
**Educational level**		
Illiterate	112	28.7
Basic education	81	20.7
Secondary education	27	6.9
Higher education	170	43.6
**Marital status**		
Married	235	60.3
Widowed	104	26.7
Divorced	47	12.1
Single	4	1.0
**Income**		
Enough	144	36.9
Not enough	246	63.1
**Dental care expenditure coverage**		
Uninsured	350	89.7
Health insurance	40	10.3
**Presence of chronic health problems ^#^**		
No	35	9.0
Hypertension	224	63.1
Diabetes mellitus	103	29.0
Respiratory diseases	49	13.8
Cardiovascular diseases	45	12.7
Gastrointestinal disorders	43	12.1
Renal disorders	41	11.5
Musculoskeletal disorders	20	5.6
Eye problems	11	3.1
Others	6	1.7
**Presence of oral health problems ^#^**		
Teeth loss	286	73.3
Teeth decay	266	68.2
Dental calculus	73	18.7
Dental plaque	41	10.5
**Smoking**		
Non- smoker	221	56.7
Smoker	111	28.5
Ex-smoker	58	14.9

^#^ Multiple response was given.

**Table 2:** Older adults’ oral health care practices, perception, dental visit satisfaction, and fear.

Variables	No. (390)	%
**Teeth brushing**		
Yes	217	55.6
No	173	44.4
**Frequency of teeth brushing/day**	**n. 217**	
Once	70	32.3
Twice	129	59.4
Three times or more	18	8.3
**Pattern of visits to the dentist**		
Emergency visits	243	62.3
Regular visits	147	37.7
**Time since last visit to the dentist**		
Within the last 6 months	61	15.6
Between 6 and 12 months	248	63.6
Between 12 and 24 months	43	11.1
More than 24 months	38	9.7
**Evaluation** **of** **the previous visits to the dentist**		
Satisfied	342	87.7
Dissatisfied	48	12.3
**Reasons for dissatisfaction in** **the previous visits to the dentist ^#^**	**n. 48**	
Long waiting time	23	47.9
Inconvenient appointments	15	31.3
Lack of respect for the elder’s dignity	13	27.1
Poor quality of the provided care	7	14.6
Ignorance of elder’s complaints by the dentist	7	14.6
Insufficient information provided by the dentist	7	14.6
**Perception of oral health value**		
Oral health is a part of the general health	304	77.9
General health is more important than oral health	72	18.5
Oral health is more important than general health	14	3.6
**Perception of oral health condition**		
Good	63	16.2
Moderate	261	66.9
Bad	66	16.9
**Reasons for dental anxiety/fear of dental visits ^#^**		
Fear of infection	269	76.2
Fear of bleeding	242	68.5
Embarrassment over the teeth shape	217	61.5
Pain felt during dental procedures	212	60.0
Fear of dental X-ray	158	44.7
Bad previous experience	50	14.2
No dental anxiety/fear	37	9.4

^#^ Multiple response was given.

**Table 3:** Oral Health Impact Profile -5 (OHIP-5) scale scores of the studied older adults.

OHIP-5	No. (390)	%
**Difficulty chewing any foods because of problems with teeth, mouth, dentures, or jaws**		
Never	56	14.4
Hardly ever	53	13.6
Occasionally	126	32.3
Often	105	26.9
Very often	50	12.8
**Painful aching in your mouth**		
Never	40	10.3
Hardly ever	64	16.4
Occasionally	146	37.4
Often	111	28.5
Very often	29	7.4
**Felt uncomfortable about the appearance of teeth, mouth, dentures, or jaw**		
Never	82	21.0
Hardly ever	32	8.2
Occasionally	149	38.2
Often	95	24.4
Very often	32	8.2
**Felt that there has been less flavor in the food because of problems with teeth, mouth, dentures, or jaws**		
Never	54	13.8
Hardly ever	38	9.7
Occasionally	119	30.5
Often	143	36.7
Very often	36	9.2
**Difficulty doing usual jobs because of problems with teeth, mouth, dentures, or jaws**		
Never	46	11.8
Hardly ever	39	10.0
Occasionally	108	27.7
Often	141	36.2
Very often	56	14.4
Overall Mean score of OHIP-5 * 15.6 ± 4.6		

* Minimum = 5 and maximum = 25, with higher numbers indicating poorer OHRQoL.

**Table 4:** Modified Dental Anxiety scale score (MDAS) of the studied older adults.

Modified Dental Anxiety Scale Score	No. (390)	%
Not anxious	37	9.5
Low anxiety	53	13.6
Moderate anxiety	39	10.0
High anxiety	107	27.4
Extreme anxiety–Phobic	154	39.5

**Table 5:** The mean score and post-hoc analysis of OHIP-5 by dental anxiety sub-groups.

MDAS Score	OHIP-5 Score		
	Mean ± SD	Significance	
		F	*p*
Not anxious a,b	13.1 ± 6.1	46.194	˂0.001 *
Low anxiety c,d	11.0 ± 4.8		
Moderate anxiety e,f	12.1 ± 4.5		
High anxiety	16.6 ± 3.4		
Extreme anxiety–Phobic	17.8 ± 2.5		

F: ANOVA test. * Significance at *p*-value ≤ 0.001. a: Not anxious vs. high anxiety (*p*-value < 0.001) b: Not anxious vs. extreme anxiety (*p*-value < 0.001). c: Low anxiety vs. high anxiety (*p*-value < 0.001) d: Low anxiety vs. extreme anxiety (*p*-value < 0.001) e: Moderate anxiety vs. high anxiety (*p*-value < 0.001) f: Moderate anxiety vs. extreme anxiety (*p*-value < 0.001).

**Table 6:** Correlation between elders’ Modified Dental Anxiety Scale (MDAS) score and Oral Health Impact Profile (OHIP-5) scale score.

	Total OHIP-5	
**Total MDAS**	r	*p*
	0.535	0.000 *

r: Pearson Correlation. *: Correlation is significant at *p* value ≤ 0.001. The absolute value of r: 0.00–0.19: “very weak”, 0.20–0.39: “weak, 0.40–0.59: “moderate”, 0.60–0.79: “strong” 0.80–1.0: “very strong”.

**Table 7:** Association between dental anxiety level and oral-health-related quality of life, controlling for sex and age.

Exposure Categories	Odds Ratio	*p*-Value	95% Confidence Interval
Dental Anxiety			
Low	3.75	0.026	1.18, 12.01
Moderate	3.03	0.066	0.93, 9.87
High	0.14	<0.001	0.06, 0.32
Extreme	0.17	<0.001	0.07, 0.38
Not anxious	1		
Sex			
Female	2.01	0.009	1.20, 3.38
Male	1		
Age			
Young old	1		
Middle old	0.622	0.516	0.16, 2.60

## Data Availability

The data that support the findings of this study are available from the corresponding author upon reasonable request.
